# Novel Wearable and Wireless Ring-Type Pulse Oximeter with Multi-Detectors

**DOI:** 10.3390/s140917586

**Published:** 2014-09-19

**Authors:** Cheng-Yang Huang, Ming-Che Chan, Chien-Yue Chen, Bor-Shyh Lin

**Affiliations:** 1 Institute of Imaging and Biomedical Photonics, National Chiao Tung University, Tainan 711, Taiwan; E-Mails: limuwaylon@gmail.com (C.-Y.H.); mingchechan@gmail.com (M.-C.C.); 2 Department of Electronics Engineering, National Yunlin University of Science and Technology, Yunlin 640, Taiwan; E-Mail: chencyue@yuntech.edu.tw; 3 Department of Medical Research, Chi-Mei Medical Center, Tainan 710, Taiwan

**Keywords:** pulse oximeter, arterial oxygen saturation, finger base, optical human tissue simulation, multi-detector

## Abstract

The pulse oximeter is a popular instrument to monitor the arterial oxygen saturation (SPO_2_). Although a fingertip-type pulse oximeter is the mainstream one on the market at present, it is still inconvenient for long-term monitoring, in particular, with respect to motion. Therefore, the development of a wearable pulse oximeter, such as a finger base-type pulse oximeter, can effectively solve the above issue. However, the tissue structure of the finger base is complex, and there is lack of detailed information on the effect of the light source and detector placement on measuring SPO_2_. In this study, the practicability of a ring-type pulse oximeter with a multi-detector was investigated by optical human tissue simulation. The optimal design of a ring-type pulse oximeter that can provide the best efficiency of measuring SPO_2_ was discussed. The efficiency of ring-type pulse oximeters with a single detector and a multi-detector was also discussed. Finally, a wearable and wireless ring-type pulse oximeter was also implemented to validate the simulation results and was compared with the commercial fingertip-type pulse oximeter.

## 1. Introduction

The pulse oximeter is a popular instrument for monitoring the arterial oxygen saturation (SPO_2_) non-invasively. By using the absorption variation of red light and near-infrared light, corresponding to the variation of the relative oxy-hemoglobin (HbO_2_) and deoxyhemoglobin (HbR) concentration in artery blood, the estimation of SPO_2_ can be calibrated by the pulse oximeter. Currently, the most popular pulse oximeter is the fingertip-type pulse oximeter [1,2]. However, the fingertip-type pulse oximeter is inconvenient for long-term monitoring, in particular, with respect to motion. Therefore, the development of wearable pulse oximeter, such as a finger-base-type pulse oximeter, can effectively improve the above issue.

In recent years, several studies have attempted to develop a finger-base-type pulse oximeter. In 2000, Yang *et al.* [3] proposed the system prototype of a ring-type pulse oximeter to monitor arterial oxygen saturation. In 2003, Asada *et al.* [4] proposed a finger base-type pulse oximeter with the transmittal illumination method and the reflective illumination method. The effect of applying a local outer pressure on the finger base was also investigated. They also indicated that the location of the light source affected the performance of measuring SPO_2_. Therefore, the complex structure of the finger base is the major problem for the design of a finger-base-type pulse oximeter. Here, the distribution of major arteries is under the phalanx. The proximal phalanx in the finger base is an opaque material, and it may obstruct most of the light penetrating through the arteries and easily affects the pathway of the penetrated light. However, there is still a lack of detailed information on the effect of the light source and the detector placement on measuring SPO_2_.

Recently, optical human tissue simulation was rapidly developed and applied in various kinds of applications. In 1993, Wang *et al.* [5] used the hybrid model of Monte Carlo simulation and diffusion theory to investigate the light reflectance in turbid media. Hiraoka [6] investigated the optical pathway length in inhomogeneous tissue and applied it in the development of near-infrared spectroscopy. In 1993, Reuss *et al.* [7] used the Monte Carlo method to investigate the design of a reflectance pulse oximeter. In 1995, Lihong Wang *et al.* [8] investigated the light transport in multi-layered tissues. In 2007, Peris *et al.* [9] used a custom Monte Carlo platform to investigate the perfusion states corresponding to different positions around the fingertip. In 2009, McEwen *et al.* [10] investigated the effect of the artery diameter variation on the function of a pulse oximeter by using the Beer–Lambert law and empirical calibration, respectively. Therefore, optical human tissue simulation can provide more valuable and reliable information to investigate the interaction between the light trace and human tissue.

In this study, the practicability of a ring-type pulse oximeter with a multi-detector was investigated by optical human tissue simulation. Here, the optical human tissue simulation was performed by using LightTools with the Monte Carlo method. Additionally, two different finger-base models were built by AutoCAD to investigate the influence of the structural differences of the finger base on the measuring performance of different ring-type pulse oximeter designs. The optimal design for the placement of the light source and detector, which can provide the best efficiency for measuring SPO_2_, was discussed. The efficiency of different ring-type pulse oximeters with a single detector and a multi-detector on measuring SPO_2_ was also discussed. Finally, wearable and wireless ring-type pulse oximeters with a single detector and a multi-detector were also implemented to validate the simulation results, and these were compared to the performance of a commercial fingertip-type pulse oximeter.

## 2. Methods and Materials

### 2.1. Fundamental Theory of Pulse Oximeter

The fundamental theory of the pulse oximeter is based on the variation of light absorption across human tissue. For the red and near-infrared wavelengths, oxyhemoglobin (HbO_2_) and deoxyhemoglobin (HbR) are the major absorbers in human tissue. By using two or more wavelengths of red and near-infrared light, the variation of light absorption, which is highly correlated to the change of the relative concentrations of HbO_2_ and HbR, and the change of the blood volume, due to the heart systole and heart diastole, can be used for the estimation of arterial oxygen saturation [11]. Here, the varying signal of light absorption related to the change of the blood volume due to the heart systole and heart diastole is called a photoplethysmograph (PPG). The pulse oximeter can calibrate and estimate SPO_2_ by the parameter of ratio R obtained from the PPG signal. Here, the parameter of ratio R [1] is defined by:

(1) $$R=\frac{AC_{R}/DC_{R}}{AC_{IR}/DC_{IR}}$$

where *AC_R_* and *DC_R_* denote the AC and DC components of the PPG signal for red light, respectively. *AC_IR_* and *DC_IR_* denote the AC and DC components of the PPG signal for near-infrared light, respectively. In this study, the parameter of ratio R was also used to evaluate the efficiency of different ring-type pulse oximeters on measuring SPO_2_. Moreover, in order to investigate the efficiency of the ring-type pulse oximeter on collecting the PPG signal, the amplitude ratio of the penetrated light to the incident light (P-I ratio) was also used to estimate the performance of the ring-type pulse oximeter design, and it was defined by:

(2) $$\text{P}‐\text{I ratio}=\frac{I_{p}}{I_{i}}\times 100\%$$

where *I_P_* denotes the intensity difference of the penetrated light between systole and diastole and *I_i_* denotes the intensity of the incident light. From the definition of the P-I ratio, a larger P-I ratio can provide better efficiency in collecting the PPG signal.

### 2.2. Optical Human Tissue Simulation

Optical human tissue simulation has been developed rapidly and applied in many applications. By using the optical properties of human tissues, optical human tissue simulation can provide more valuable and reliable information. Additionally, the Monte Carlo method is most frequently used for ray tracing in optical human tissue simulations [12]. In this study, the Monte Carlo method was also used to perform the optical human tissue simulation to investigate the design of the ring-type pulse oximeter. Here, the Henyey–Greenstein phase function was also used to describe the scattering behavior [13] and is given by:

(3) $$P_{HG}(\theta )=\frac{w(1-g^{2})}{{\left(1+g^{2}-2g\cos(\theta )\right)}^{\frac{3}{2}}}$$

where *P_HG_* denotes the intensity in a specific degree and *w* and *θ* denote a normalization parameter and the direction of scattering, respectively. The phase function represents the power distribution of scattering from 0° to 180°. Here, the scattering anisotropy parameter (*g*) [13] is given by:

(4) $$g=\langle \cos\theta \rangle =\int_{0}^{\pi }P_{HG}(\theta )\cos\theta 2\pi \sin\theta d\theta$$

The used optical properties of human tissues in the optical human tissue simulation, including the absorption coefficient (μa), the scattering coefficient (μs), the refractive index (n), the mean free path (MFP) and the anisotropy parameter (g), are shown in Table 1 [10,14–18]. The probability of the reflectance and transmittance for Lambertian scattering are shown in Table 2 [19–22].

**Figure 1.**
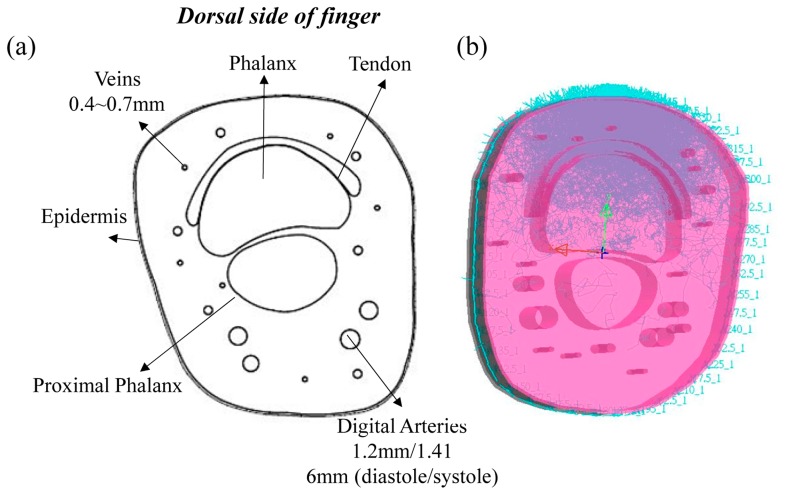
(**a**) Cross-section of the finger base and (**b**) the optical human tissue simulation when the light source was placed at 0°.

The structure of the finger base is shown in Figure 1a [23,24], and it includes the tissue components of the epidermis, dermis, phalanx, proximal phalanx, digital arteries and veins. Based on the structure of the finger base, two different finger-base models were built by AutoCAD. Here, the thickness of epidermis is about 0.2 mm, and the thickness of the finger base is about 20 mm. A pair of digital arteries were set under the proximal phalanx, and several veins were placed around the phalanx. The diameter of the diastole digital arteries is about 1.2 mm, and that of the systole digital arteries is about 1.416 mm. The diameter of several veins is about 0.4∼0.7 mm. The sizes of two finger-base models are 40 mm × 18.4 mm × 22.4 mm and 40 mm × 22.3 mm × 25.8 mm, respectively. Certainly, the tissue shape, the size and the placement of tissues in the finger base are also different. Figure 1b shows the randomly selected optical human tissue simulation result. Additionally, each finger model contains two modes (the diastolic and systolic modes). The difference between the diastolic and systolic modes of each finger model is only the diameter of the digital arteries, and the simulation results of the diastolic and systolic modes were used to calculate the parameter R and the P-I ratio.

### 2.3. Implementation of the Wearable and Wireless Ring-Type Pulse Oximeter

**Figure 2.**
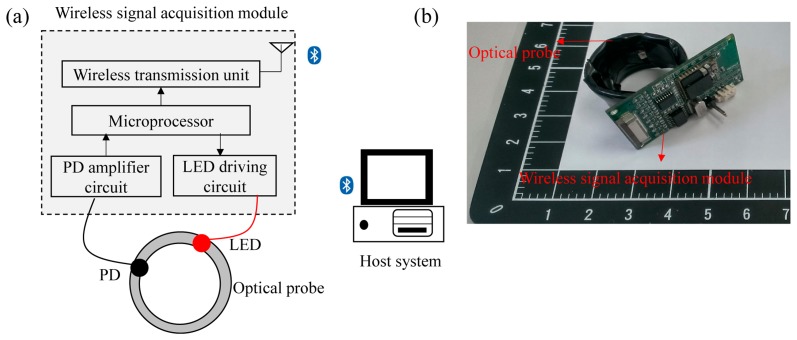
(**a**) Block diagram and (**b**) photograph of the wearable and wireless ring-type pulse oximeter. PD, photodiode.

**Figure 3.**
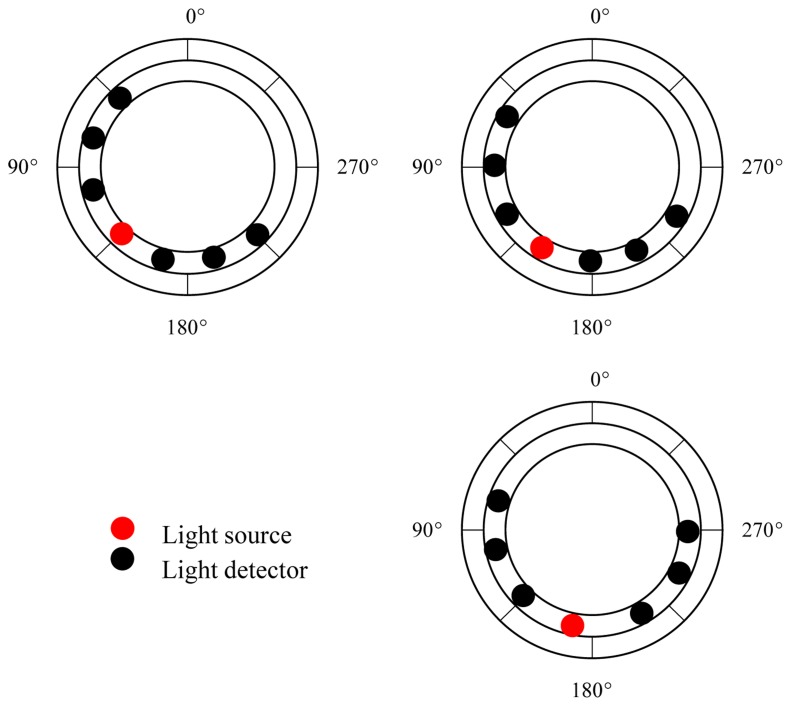
Three types of optical probe placements. The light source was placed at 135°, 150° and 165°, respectively.

In order to validate the simulation results, a wearable and wireless ring-type pulse oximeter was designed and implemented in this study. The basic block diagram of the ring-type pulse oximeter is shown in Figure 2. It mainly contains an optical probe and a wireless signal acquisition module. Here, the optical probe was designed to provide the light source and receive the penetrated light that passes through the tissue. The surface-mounted device (SMD) light-emitting diode (LED; SMT660/940, epitex, Japan) and silicon-pin photodiode (PD; PD15-22C/TR8, EVERLIGHT, Taiwan) were embedded in the optical probe and used as the light source and the light detector. The LED used can provide the 660-nm and 940-nm light sources. In order to investigate the efficiency of different ring-type pulse oximeter designs, three types of optical probes were assembled. Additionally, the placements of the light source and the light detectors for the three optical probes are shown in Figure 3.

The wireless signal acquisition module mainly contains a microprocessor, an LED driving circuit, PD amplifier circuits and a wireless transmission unit. Here, the microprocessor (MSP430, Texas Instruments, USA) was designed to control the LED driving circuit to turn on or turn off the LED. Next, the penetrated light will be received, amplified and filtered by PD amplifier circuits. Here, the PD amplifier circuits contain 20 M trans-resistance amplifiers to convert the output current of the light detector into a voltage signal. Then, the amplified penetrated light signal will be digitized by a 12-bit analog-to-digital converter (ADC) built in the microprocessor with a sampling rate of 25 Hz. Finally, the digitized penetrated light signal will be sent to the wireless transmission unit to transmit to the host system wirelessly. The wireless transmission unit consists of a Bluetooth module with Bluetooth v2.0 compliant specification and a printed circuit board (PCB) antenna. The monitoring program built in the host system will receive, display, store and analyze the penetrated light signal.

## 3. Results

### 3.1. Simulation Results

**Figure 4.**
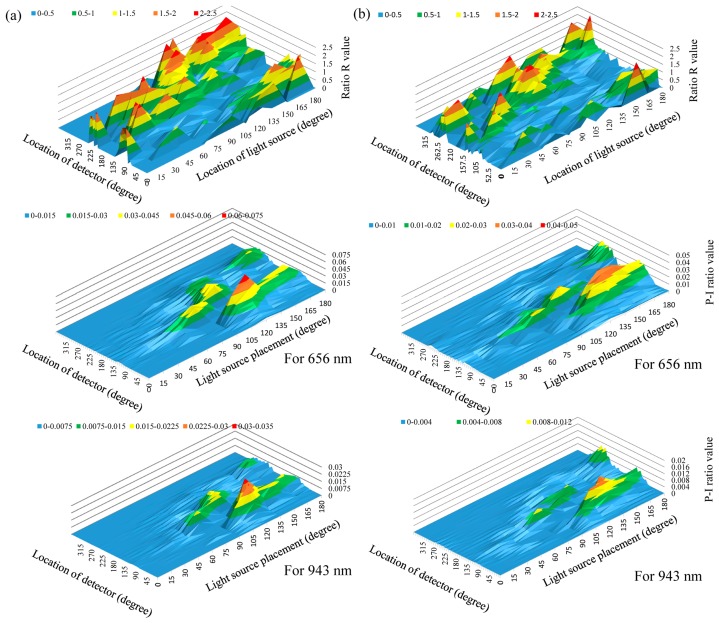
R ratio values and P-I (penetrated light to the incident light) ratio values of (**a**) Model 1 and (**b**) Model 2, corresponding to different locations of light sources and detectors.

In this section, the efficiency of different ring-type pulse oximeter designs with a single detector was first investigated. The light source was placed at 0°, 15°, 30°, 45°, 60°, 75°, 90°, 105°, 120°, 135°, 150°, 165° and 180°, respectively, and here, 0° denotes the top of the finger base in this study. Additionally, a total of 48 light detectors were also equiangularly placed around the finger base. In this simulation, the number of photons was set to ten million. Figure 4 shows the values of ratio R and the values of Models 1 and 2 corresponding to different locations of the light source and the detector. Here, the value of ratio R was set to zero, if the value of ratio R were unreasonable (>2.5).

The simulation result shows that the values of the P-I ratio are very low when the light source was placed at 15°–30°. This also indicated that it is difficult for the light detector to collect the penetrated light when the light source is placed at 15°–30°. When the light source was placed at 45°–60°, placing the detector at 90°–120° can provide a relatively larger P-I ratio value than that of 15°–30°, but these P-I ratio values are still not sufficient. When the light source and the detector were placed at 75°–90° and 105°–135°, respectively, or they were placed at 105°–120° and 130°–150°, these designs can provide better P-I ratio values. However, for these design, the variation of the P-I ratio value is easily affected by the change of the finger base architecture. When the light source was placed at 135°–165°, placing the detector at 75°–90° or at 90°–105° can provide better P-I ratio values and reasonable and stable ratio R values. Therefore, placing the light source at 135°–165° and placing the detector at 75°–90° or at 90°–105° is a good design for a ring-type pulse oximeter with a single detector. When the light source was placed at 180° and the detector was placed at 105°–120° or at 240°–255°, these designs can also provide relatively larger P-I ratio values, but are still smaller than that of the light source at 135°–165°.

**Figure 5.**
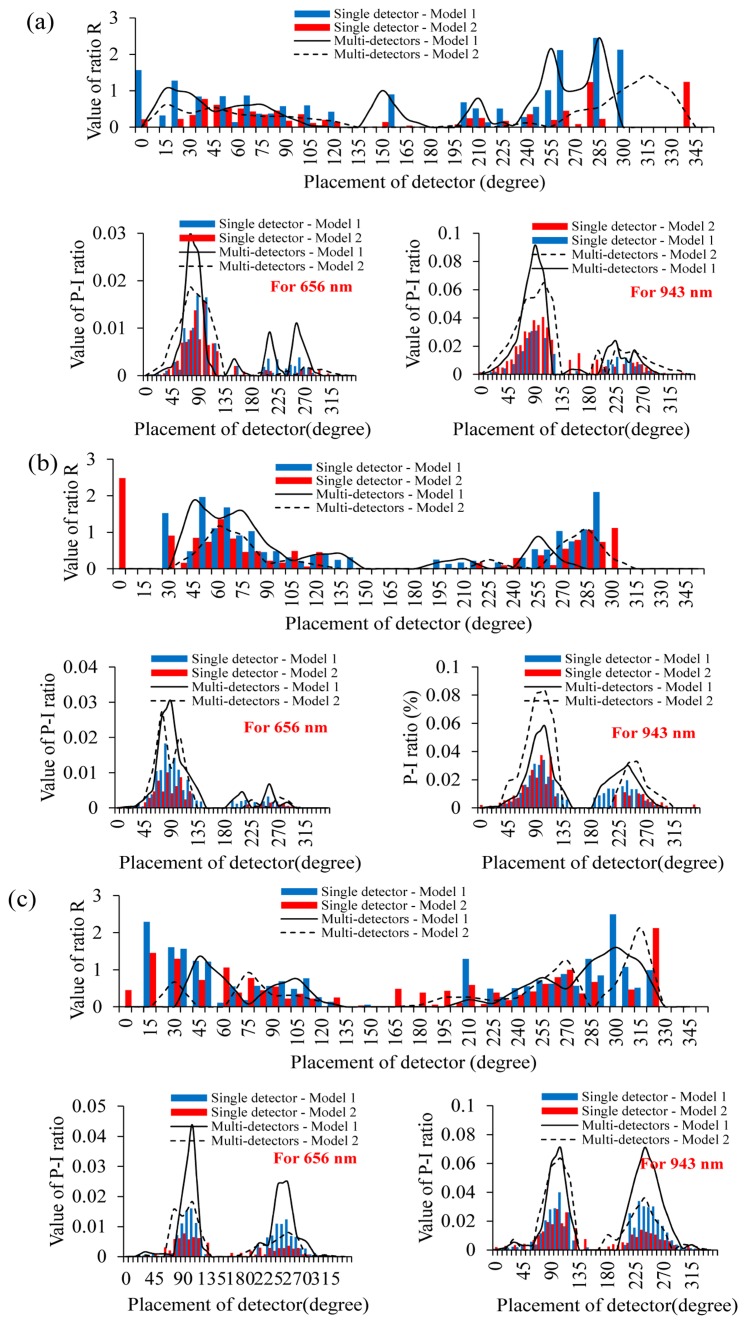
Comparisons of simulation results for the P-I ratio value and ratio R value by using a single detector and a multi-detector when the light source was placed at (**a**) 135°, (**b**) 150° and (**c**) 165°, respectively.

Next, the efficiency of different ring-type pulse oximeter designs with a multi-detector was investigated. Here, the detector array consisting of three light detectors was used as the multi-detector. From the simulation results of the ring-type pulse oximeter with a single detector, these designs for placing the light source at 135°–165° can provide better P-I ratio values and reasonable ratio R values. Therefore, for this simulation, the light source was also placed at 135°–165°. Figure 5 is the comparison of the simulation results of P-I ratio values and ratio R values for a single detector and a multi-detector when the light source was placed at 135°, 150° and 165°, respectively. Compared with the result of using the single detector, the variation of ratio R values corresponding to different detector locations for a multi-detector become more stable. Therefore, for a multi-detector, the influence of selecting the detector location on the performance of measuring SPO_2_ becomes small. Moreover, the P-I ratio values for these designs (the light source at 135°, the multi-detector at 75°–105°; the light source at 150°, the multi-detector at 90°–105°; the light source at 165°, the multi-detector at 90°–105° or at 240°–255°) can be effectively enhanced.

### 3.2. Experimental Results

**Figure 6.**
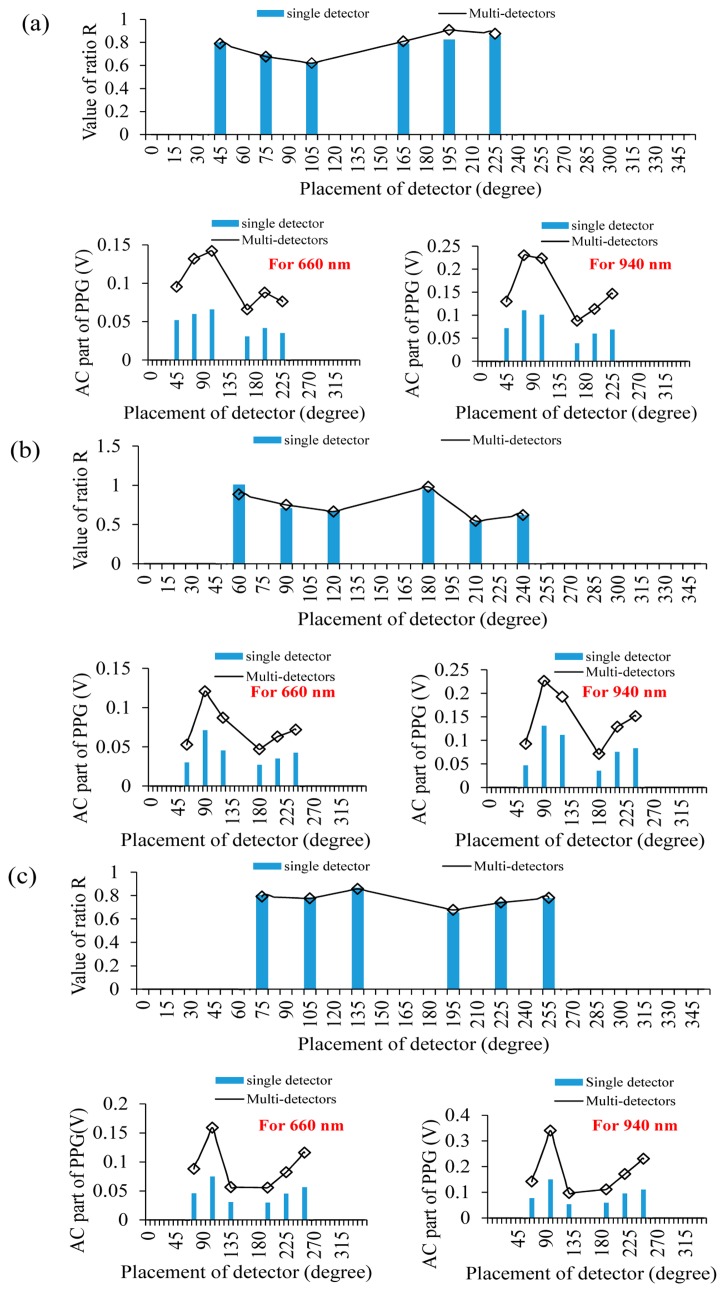
Comparisons of the experimental results for the AC part of the photoplethysmograph (PPG) and ratio R values by using a single detector and a multi-detector when the light source was placed at (**a**) 135°, (**b**) 150° and (**c**) 165°, respectively.

In this section, a wearable and wireless ring-type pulse oximeter was implemented to validate the simulation results. The light source of the optical probe was placed at 135°, 150° and 165°, respectively. Figure 6a, Figure 6b and Figure 6c show the comparisons of the measured ratio R values and the AC part of the PPG signal for the single detector and multi-detector when the light source was placed at 135°, 150° and 165°, respectively. When using the single detector, when the light source was placed at 135°, placing the detector at 75° can provide the larger AC part of the PPG signal and a reasonable ratio R value. When the light source was placed at 150° and the detector was placed at 90°, the AC part of the PPG signal is obviously larger than other detector locations, and the ratio R value was also reasonable. Similarly, when the light source was placed at 165°, placing the detector at 105° provided the larger AC part of the PPG signal and a reasonable ratio R value. The above experimental results exactly fit the simulation results.

When using the multi-detector, when the light source was placed at 135°, placing the multi-detector at 75° can provide the larger AC part of the PPG than that of using a single detector, and the ratio R value is also reasonable. When the light source and the multi-detector were placed at 150° and 90°, respectively, and the light source and the multi-detector were placed at 165° and 105°, respectively, these designs can also provide the larger AC part of the PPG than that using the single detector. Therefore, using the multi-detector can enhance the efficiency of collecting the PPG signal and reduce the influence of selecting the detector location.

**Figure 7.**
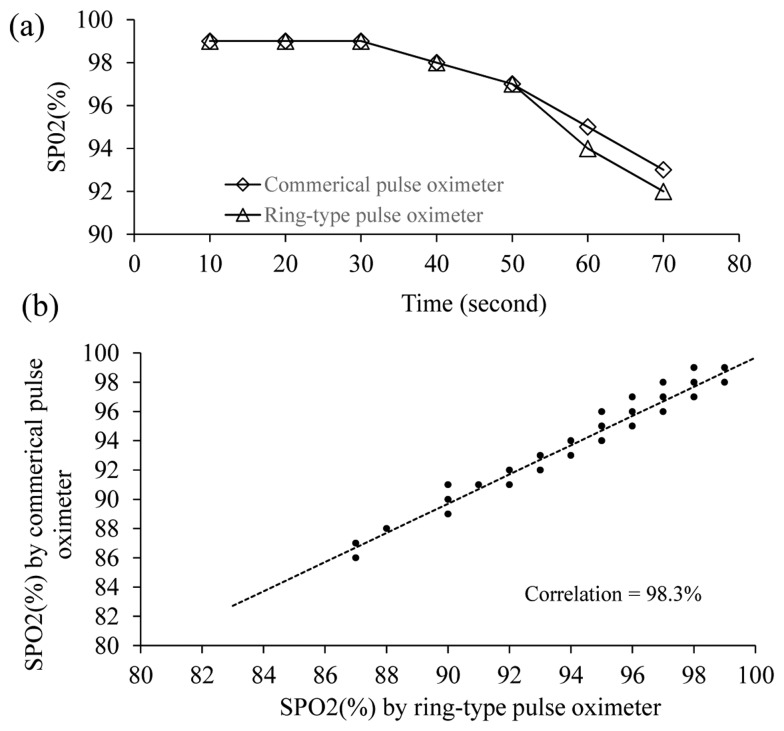
(**a**) Experimental results of measuring SPO_2_ by different pulse oximeters; (**b**) correlation between SPO_2_ measured by the ring-type pulse oximeter and the commercial fingertip-type pulse oximeters.

Next, the performance of the proposed ring-type pulse oximeter with the multi-detector (the light source is at 165° and the detector is at 105°) for measuring SPO_2_ was compared with that of a commercial fingertip-type pulse oximeter (DB11, DELBIO, Taiwan). A total of 10 healthy participants joined this experiment. The participant was instructed to equip themselves with the proposed ring-type pulse oximeter and the commercial fingertip-type pulse oximeter simultaneously. Next, the participant was instructed to hold his/her breath for about 70 s. Figure 7 shows one of the experimental results for the change of SPO_2_ measured by different pulse oximeters. It shows that both of the SPO_2_ values measured by different pulse oximeters dropped slowly from 99% to 97% during the first 50 s, and dropped drastically from 97% to 92% after 50 s. The correlation between the SPO_2_ values measured by the proposed ring-type pulse oximeter and the commercial fingertip-type pulse oximeter is high (98.26%). Therefore, the proposed ring-type pulse oximeter with the multi-detector can provide similar performance to the commercial fingertip-type pulse oximeter.

## 4. Discussions

From the simulation results, when the light source was placed at 0°–30°, the P-I ratio was very small and the value of the R ratio was unstable. This is due to the light source being placed at the opposite site of the digital arteries and the phalanx. The phalanx is the major opaque material obstructing most of the penetrated light. When the light source was placed at 45°–120°, the P-I ratio can be improved and was larger than that of placing the light source at 0°–30°. However, the light source in these design is close to the phalanx. Much of the penetrated light is easily affected by the shape, size and location of the phalanx. Therefore, for different finger-base models, the value of the P-I ratio is easily affected by the structural differences of the finger base.

When the light source was placed at 135°–165° and the detector placed at 75°–105°, most of the photons pass through the digital arteries. Moreover, the light source and detector were placed at the same side of the digital arteries. Therefore, the influence of the phalanx structural differences on the performance of collecting the PPG signal is small. Compared with the performance of placing the light source at 135°–165° and the detector at 75°–105°, the efficiency of collecting the PPG for placing the light source at 180° and the detector at 105°–120° or at 240°–255° is poorer. This can be explained by the penetrated light having to pass through a longer pathway to the detector. Therefore, for this design, the value of ratio R is reasonable, but the P-I ratio is relatively small.

The unreasonable R ratio values mainly resulted from the very small ratio of *AC_IR_* to *DC_IR_*. This is due to that the phalanx obstructing the penetrated light, or the penetrated light did not pass through the digital arteries. This phenomenon can also be reflected in the distribution of the P-I values and the location of the light source. Therefore, when the P-I value is larger and the light source is also close to the digital arteries, this easily provides a stable R value.

## 5. Conclusions

In this study, the optimal design of a ring-type pulse oximeter for the light source and detector placement was investigated. From the simulation results, the ring-type pulse oximeter design of placing the light source at 135°–165° and the detector at 75°–90° or at 90°–105° can provide better P-I ratio values and stable ratio R values. Moreover, using the multi-detector can enhance the stability and efficiency of collecting the PPG signal. Moreover, a wearable and wireless ring-type pulse oximeter was also designed and implemented. From the experimental results, it is indicated that using a multi-detector can enhance the efficiency of collecting the PPG signal. Finally, the performance of the proposed ring-type pulse oximeter with multi-detector was compared with that of the commercial fingertip-type pulse oximeter. From the experimental results, the correlation between the SPO_2_ values obtained by the proposed ring-type and the commercial fingertip-type pulse oximeters is high.

## Figures and Tables

**Table 1.** Optical properties of tissues in simulation.

		**Dermis and Pulp**	**Epidermis**	**Bone**	**Arterial Blood (97% SaO_2_)**	**Venous Blood (60% SvO_2_)**
μa (mm^Minus1^)	656 nm	0.05	0.05	0.05	0.2	0.72
	943 nm	0.03	0.03	0.02	0.64	0.54

μs (mm^Minus1^)	656 nm	8.6	18.3	33.1	77.5	77.5
	943 nm	7	12.3	26.1	65	65

n	656 nm	1.38	1.43	1.4	1.363	1.362
	943 nm	1.38	1.42	1.4	1.358	1.357

MFP (mm)	656 nm	0.1156	0.0545	0.0301	0.0129	0.0128
	943 nm	0.1422	0.0811	0.0383	0.0152	0.0153

g	656 nm	0.91	0.81	0.92	0.98	0.98
	943 nm	0.91	0.89	0.94	0.99	0.99

**Table 2.** Lambertian scattering parameters for the finger-base model.

**Lambertian Scattering**	**Epidermis**	**Dermis**	**Veins**	**Phalanx**	**Proximal Phalanx**	**Digital Arteries**
Reflectance	32%	26%	34%	47%	26%	53%
Transmittance	68%	74%	66%	53%	74%	47%

## References

[1] Petersen C.L., Chen T.P., Mark Ansermino J., Dumont G.A. (2013). Design and Evaluation of a Low-Cost Smartphone Pulse Oximeter. Sensors.

[2] Rubortone S.A., De Carolis M.P., Lacerenza S., Bersani I., Occhipinti F., Romagnoli C. (2012). Use of a Combined SpO_2_/PtcCO_2_ Sensor in the Delivery Room. Sensors.

[3] Yang B.H., Rhee S. (2000). Development of the ring sensor for healthcare automation. Robot. Auton. Syst..

[4] Asada H.H., Shaltis P., Reisner A., Rhee S., Hutchinson R.C. (2003). Mobile monitoring with wearable photoplethysmographic biosensors. IEEE Eng. Med. Biol. Mag..

[5] Wang L., Jacques S.L. (1993). Hybrid model of Monte Carlo simulation and diffusion theory for light reflectance by turbid media. J. Opt. Soc. Am..

[6] Hiraoka M., Firbank M., Essenpreis M., Cope M., Arridge S.R., van der Zee P., Delpy D.T. (1993). A Monte Carlo investigation of optical pathlength in inhomogeneous tissue and its application to near-infrared. Phys. Med. Biol. Inst..

[7] Reuss J.L. (2005). Multilayer Modeling of Reflectance Pulse Oximetry. IEEE Trans. Biomed. Eng..

[8] Wang L., Jacques S.L., Zheng L. (1995). MCML-Monte Carlo modeling of light transport in multi-layered tissues. Comput. Methods Progr. Biomed..

[9] Peris V.A., Hu S. (2007). Validation of a Monte Carlo platform for the optical modelling of pulse oximetry. J. Phys..

[10] McEwen M.P., Bull G.P., Reynolds K.J. (2009). Vessel calibre and haemoglobin effects on pulse oximetry. Physiol. Meas..

[11] Tsai H.-Y., Huang K.-C., Chang H.-C., Yeh J.-L.A., Chang C.-H. (2014). A Noncontact Skin Oxygen-Saturation Imaging System for Measuring Human Tissue Oxygen Saturation. IEEE Trans. Instrum. Meas..

[12] Zhu C., Liu Q. (2012). Hybrid method for fast Monte Carlo simulation of diffuse reflectance from a multilayered tissue model with tumor-like heterogeneities. J. Biomed. Opt..

[13] Tuchin V.V. (1997). Light scattering study of tissues. Phys. Uspekhi.

[14] Netz U.J., Scheel A.K., Beuthan J., Hielscher A.H. Development of a finger joint phantom for evaluation of frequency domain measurement systems.

[15] Bashkatov A.N., Zhestkov D.M., Genina E.A., Tuchin V.V. (2005). Immersion clearing of human blood in the visible and near-infrared spectral regions. Opt. Spectrosc..

[16] Ding H.F., Lu J.Q., Wooden W.A., Kragel P.J., Hu X.H. (2006). Refractive indices of human skin tissues at eight wavelengths and estimated dispersion relations between 300 and 1600 nm. Phys. Med. Biol..

[17] Faber D.J., Aalders M.C., Mik E.G., Hooper B.A., van Gemert M.J., van Leeuwen T.G. (2004). Oxygen saturation-dependent absorption and scattering of blood. Phys. Rev. Lett..

[18] Zhernovaya O., Tuchin V.S., Douplik A. (2011). The refractive index of human hemoglobin in the visible range. Phys. Med. Biol..

[19] Locking P.M., Banks D.P. A Simple Source Temperature Dependent Human Burn Injury Model.

[20] Nielsen K.P., Zhao L., Stamnes J.J., Stamnes K., Moan J. (2008). The optics of human skin: Aspects important for human health. Nor. Acad. Sci. Lett..

[21] Doemer B., Klausmann D., Bindig U., Gersonde I., Schwaibold M., Schoeller B., Kotterba B. Measurement of Optical Pulsation and Transmission Spectra as Reference for a Monte Carlo Simulation of the Finger Tip.

[22] Bashkatov A.N., Genina É. A., Kochubey V.I., Tuchin V.V. (2005). Optical properties of the subcutaneous adipose tissue in the spectral range 400–nm. Opt. Spectrosc..

[23] Krawiecki Z., Cysewska-Sobusiak A., Wiczynski G., Odon A. (2008). Modeling and measurements of light transmission through human tissues. Bull. Polish Acad. Sci. Tech. Sci..

[24] Erickson S.J., Kneeland J.B., Middleton W.D., Jesmanowicz A., Hyde J., Lawson T.L., Foley W.D. (1989). MR imaging of the finger: correlation with normal anatomic sections. Am. J. Roentgenol..

